# The effect of NaOH pretreatment on coal structure and biomethane production

**DOI:** 10.1371/journal.pone.0231623

**Published:** 2020-04-15

**Authors:** Hongguang Guo, Xingfeng Li, Jinlong Zhang, Zaixing Huang, Michael A. Urynowicz, Weiguo Liang

**Affiliations:** 1 College of Safety and Emergency Management and Engineering, Taiyuan University of Technology, Taiyuan, Shanxi, China; 2 Key Lab of In-situ Property-improving Mining of Ministry of Education, Taiyuan University of Technology, Taiyuan, Shanxi, China; 3 School of Chemical Engineering and Technology, China University of Mining and Technology, Xuzhou, Jiangsu, China; 4 Center for Biogenic Natural Gas Research, Department of Civil and Architectural Engineering, University of Wyoming, Laramie, Wyoming, United States of America; 5 College of Mining Engineering, Taiyuan University of Technology, Taiyuan, Shanxi, China; China University of Mining and Technology, CHINA

## Abstract

Biogenic CBM is an important component of detected CBM, which is formed by coal biodegradation and can be regenerated by anaerobic microorganisms. One of the rate-limiting factors for microbial degradation is the bioavailability of coal molecules, especially for anthracite which is more condense and has higher aromaticity compared with low-rank coal. In this paper, NaOH solution with different concentrations and treating time was employed to pretreat anthracite from Qinshui Basin to alter the coal structure and facilitate the biodegradation. The results showed that the optimal pretreatment conditions were 1.5 M NaOH treating for 12 h, under which the biomethane production was increased by 17.65% compared with untreated coal. The results of FTIR and XRD showed that NaOH pretreatment mainly reduced the multi-substituted aromatics, increased the C-O in alcohols and aromatic ethers and the branching degree of aliphatic chain, and decreased the aromatic ring structure, resulting in the improvement of coal bioavailability and enhancement of biomethane yield. And some organics with potential to generate methane were released to filtrate as revealed by GC-MS. Our results suggested that NaOH was an effective solution for pretreating coal to enhance biogenic methane production, and anthracite after treating with NaOH could be the better substrate for methanogenesis.

## Introduction

The exploitation and utilization of coalbed methane (CBM) can improve the energy structure worldwide ensuring the sustainable development of energy. It also can greatly reduce the cost of coal mining as methane is the main hazard in the coal mine. Biogenic CBM is an important part of detected CBM, which has been found in many CBM fields around the world using isotope techniques [1,2]. It is believed that biogenic CBM is formed by coal biodegradation, which makes it possible to regenerate.

Until now, a number of studies have demonstrated that coal in different ranks can be degraded to produce methane by indigenous and exogenous microorganisms [3,4]. Living microbial communities from Well-bore water in the Powder River Basin of Wyoming have been found to generate methane by degrading Wyodak coal under laboratory conditions [3]. A set of bioassay method was developed to estimate the potential of coal samples for methane production under defined laboratory conditions [4]. Six lignite samples were observed to generate methane by a mixed methanogenic consortium (WBC-2) under anaerobic conditions [5]. It was demonstrated that microbes were capable of converting coal into methane, and the methane yield increased 24.3 times when the homemade water-based recipe was added [6]. The fact that coal can be degraded by indigenous microorganisms to produce methane was also demonstrated through pilot-scale fermentation of lump anthracite [7].

Coal biodegradation is mainly carried out by anaerobic microbes which transformed macromolecular organic compounds in coal into smaller organic molecules and then further converted to methane [1,8]. However, due to the complex molecular structure of coal, it is difficult for microorganisms to act directly on the macromolecular structure and degrade it, resulting in lower gas production and longer period. Only about 0.1–22.9 mmol of biomethane can be produced from 1 gram coal according to the previous studies [9]. The rate-limiting step of microbial degradation of coal is thought to be the fracturing of coal macromolecules in the early stage of coal degradation [10]. Thus, several pretreatment methods have been proposed, e.g. chemical treatment, physical treatment, fungal treatment, and bacterial treatment [10–13], to decrease the complexity of coal and increase the bioavailability of coal, thereby enhancing the bioconversion of coal. Chemical pretreatment is widely studied in the biotransformation of coal. The reported chemical reagents include potassium permanganate (KMnO_4_) [14], hydrogen peroxide (H_2_O_2_) [15–17], nitric acid (HNO_3_) [18,19], sodium hydroxide (NaOH) [20,21], and so on. These reagents are proven to enhance methane production by increasing the solubility of organic carbon in coal, especially subbituminous coal and lignite. For example, each gram of subbituminous coal can produce 93.4 μmol methane in 40 days after treating with KMnO_4_ [14].

During the growth of microorganisms, some alkaline substances, such as ammonium ions and biogenic amines, could be secreted or produced by utilizing certain compounds in the environment. These alkaline substances might participate in the degradation of coal. Strandberg reported in l987 that low-ranked coal could be solubilized by an extracellular substance produced by *Actinomyces* and the amount of liquefied coal increased with the increase of the culture media pH which is related to the amount of polypeptides and polyamines contained in it [22]. In 1989, Quigley reported that an alkaline metabolite produced by fungi could ionize the acidic functional groups of low-ranked coal, thus improving the hydrophilicity of coal [23]. In the experiment of lignite biodegradation with crude oil hydrocarbon-degrading bacteria, the extracellular solubilization activities of tested bacteria decreased with the decrease of pH, indicating the effect of some alkaline chemicals on the process of coal liquefaction [24]. Moreover, it was proved that alkali treatment could break, disrupt, and reform various interaction forces and entanglements between coal macromolecules [25]. And NaOH participates in the reaction system of liquefaction of lignite, splitting the coal structure [26]. Therefore, the pretreatment of coal with NaOH could change the organic structure in coal, which is conducive to biodegradation. Moreover, NaOH is easily to be transformed into sodium salt which is also required by the growth of microorganisms. In addition, the salinities of produced water from CBM fields are generally high [27,28].

In fact, NaOH pretreatment has been reported to increase the solubility of coal. Huang et al. treated subbitumious with NaOH, and found the enhancement of solubility of organic carbon and the releasement of aromatic fragments with low molecular weight [20,21]. Most reported chemical pretreatments including NaOH pretreatment were performed on low-rank coal or bituminous coal, while anthracite has rarely been reported. On the contrary, anthracite is more condense and has higher aromaticity than those low-rank coals [29] so that it is difficult for microbial degradation which makes it urgently to explore effective chemical pretreatment methods to increase the bioavailability of anthracite and the biomethane production from anthracite. In addition, the effect of NaOH pretreatment on coal structure and the relationship between structural changes and methane production are not clear yet. Thus, an experiment was performed to pretreat anthracite using different concentrations of NaOH for varied treating time to determine the effect of NaOH pretreatment on methane production and the optimal treating conditions. And the mechanism of enhancing biomethane by NaOH pretreatment was also analyzed from the aspects of coal structure and soluble organics by Fourier-transform infrared spectroscopy (FTIR), X-ray diffraction (XRD), and GC-MS.

## Materials and methods

### Coal sample and microflora

The coal sample was collected from Sihe coal mine in Qinshui basin, south-central Shanxi province, where biogenic CBM has been found and diverse microbial communities have been detected in the produced water [30]. We state no specific permissions were required for these locations/activities. We confirm the field studies did not involve endangered or protected species. The sample was ground into a powder (120 mesh) and dried at 45 °C for 6 h before used. The proximate analysis (M_ad_, A_d_, V_daf_) and ultimate analysis (C, H, O, N, S) are performed respectively according to national standards of GBT212-2008 and GB476-91. The corresponding results are as follows: moisture, 1.90%; ash, 10.41%; volatiles, 8.82%; C, 83.01%; H, 3.24%; O, 1.76%; N, 1.27%; S, 0.31%.

The microflora QSB-1 used in the experiment was enriched from produced water obtained from the same site as the coal sample. It is capable of biodegrading coal to produce methane under anaerobic conditions with optimum growth temperature at 35 °C.

### NaOH pretreatment of coal

Three concentrations of NaOH including 0.1 M, 0.5 M, and 1.5 M were selected for pretreating the coal. The dried coal sample (g) and NaOH solution (ml) were mixed at a ratio of 1:5 and stirred by a magnetic stirrer for 6 h. Then, the mixtures were filtered using a 0.22 μm filter (Millipore, USA), and washed with sterilized water until the filtrate became neutral. The residual coal samples on the filter were dried at 45°Cfor 6h and weighed before anaerobic cultivation.

Based on the results of methane production in cultivations with pretreated coal samples, a proper concentration of NaOH would be determined. Under the selected concentration of NaOH, different pretreating time including 4 h, 8 h, 12 h, and 16 h was chosen to determine the optimal treating time. The detailed procedures were the same as the previous experiment of different concentrations.

### Anaerobic cultivation and methane production

The cultivations were carried out in 140 ml culture bottles. 1.0 g pretreated coal or raw coal was loaded in each bottle with 27 ml anaerobic medium and 3 ml microflora QSB-1. The anaerobic medium included 3 mL basic medium, 0.15 mL trace metals solution, 0.15 mL vitamin solution, 0.6 mL cysteine (15%)—Na_2_S (15%), and 23.1 mL sterile water. The composition and content of the basic medium, trace mineral solution, and vitamin solution were prepared according to our previous work [31]. The cultivations without coal were setup as negative control. All the experiments were carried out in triplicate and all the bottles were incubated without shaking at 35 °C. Methane production in the headspace of the bottles was periodically determined.

### FTIR analysis

FTIR was employed to analyze the changes of functional groups after NaOH treatment. FTIR analysis was performed according to the description in previous literature [32]. 0.001 g coal sample and 0.1 g KBr were ground in a mortar until they were mixed completely. Then the mixture was pressed into pellets by keeping 1 min under a pressure of 10 MPa. The FTIR analysis was implemented using a Bruker Tensor 27 Fourier-transform infrared spectrometer, and the spectra of 64 scans were accumulated at a resolution of 4 cm^-1^. The prepared pellets were detected by the spectrometer, and the measured wavenumbers region ranged from 400 to 4000 cm^-1^. The correlative data were obtained with the built-in operation software OPUS 5.5 and analyzed using Origin 8.0 software. And curve-fitting analysis was further performed using the Peakfit 4.12 software in the selected region of obtained spectrograms to obtain the detailed peak positions, intensities, widths, and areas.

### XRD analysis

The microcrystalline structure of the coal sample was determined by XRD. A TD-3500 X-ray diffractometer was adopted to collect XRD data from the obtained coal samples with Cu Ka radiation under the condition of 30 kV and 20 mA. The continuous scanning mode was used in a range of 5 to 90° with a 0.05° step interval and a 6°/min scanning rate. Jade software was used to fit the diffraction patterns in the two regions of 16–34° and 39–49°. The broad hump in the region of 16–49° was fitted to two Gaussian peaks around 26° and 43°, representing the peak 002 and 100, respectively [33]. The positions, intensities, widths, and areas of the corresponding peak were also determined.

The microcrystalline structure parameter L_a_ and L_c_ was determined according to conventional Scherrer equations [34,35]: L_a_ = 1.84λ/(β_a_ cos φ_a_) and L_c_ = 0.94λ/(β_c_ cos φ_c_), where λ is the wavelength of X-ray; β_a_ and β_c_ are the half width of peak 100 and peak 002; φ_a_ and φ_c_ are the corresponding scattering angles, respectively. The aspect ratio of the stacking aromatic layer is equal to the ratio of L_a_ to L_c_. The stacking layer number (N) of aromatic carbon corresponds to the ratio of L_c_ to d_002_, where d_002_ can be computed by λ/2sinφ_c_.

### GCMS analysis

The filtrate obtained after pretreatment with 1.5 M NaOH for 12 h was extracted using 10 mL CH_2_Cl_2_ for three times at neutral, alkaline, and acidic conditions, respectively. All the separated liquid was mixed. Excess anhydrous sodium sulfate was added in the mixed liquid, and the sealed bottle was placed at 4 °C for 12 h before rotary evaporation at a temperature of 40 °C. The obtained solution was evaporated to about 5 mL and concentrated to about 1 mL by nitrogen purge. Then, the organics in the extracted samples were analyzed by GC-MS system (7890B/5977B, Agilent, USA). The GC column was operated in a temperature programmed mode by maintaining the temperature at 60 °C for 3 min, then increasing to 150 °C with an increment of 20 °C/min, finally increasing to 230 °C with an increment of 5 °C/min and then maintaining for 5 min. The identification of the organic compounds was undertaken with reference of National Institute Standard and Technology (NIST 14L).

## Results and discussions

### Effects of NaOH concentration on methane production

**Fig 1:**
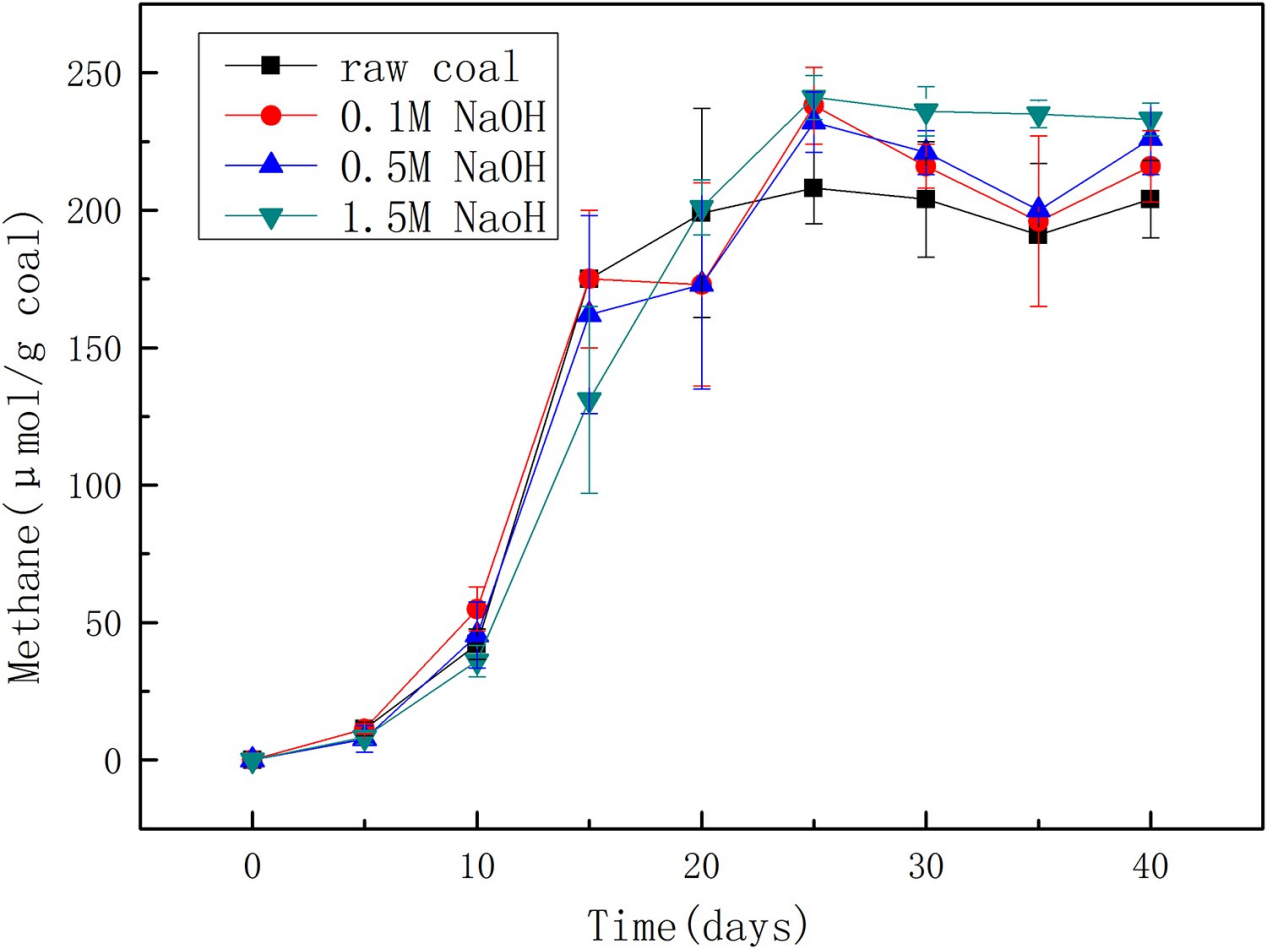
Methane productions from coal pretreated by 0.1 M, 0.5 M, and 1.5 M NaOH.

In order to study the effect of NaOH concentration on biomethane production, the pretreatment of coal was implemented by NaOH with different concentrations. After about 40 days of cultivation, significant methane was produced from both raw coal and pretreated coal (Fig 1). After treating by 0.1 M NaOH, methane production reached 216 μmol/g coal which was 5.88% higher than that produced from the raw coal with 204 μmol/g coal. 0.5 M NaOH pretreatment increased methane production by 10.78% with 226 μmol/g coal, while 1.5 M NaOH pretreatment had the largest methane yield with 233 μmol/g coal which was 14.22% higher than that produced from the raw coal. The methane productions from pretreated coal using different concentrations of NaOH were all larger than that from raw coal, indicating that NaOH pretreatment has a positive effect on the production of biomethane by changing coal structure to facilitate coal biodegradation. And the final methane production at the end of cultivation increased with the increase of NaOH concentration, suggesting that the higher the concentration of NaOH was, the greater the changes of coal were, and the changes of coal structure caused by NaOH pretreatment became more conducive to microbial action with higher concentration.

The methane production rates in cultivations with coal pretreated by different concentrations of NaOH have the same trend, which can be briefly divided into three periods. The first 10 days is a slow growth period (lag phase) when methane production is relatively slow, which may be due to the low abundance of microflora. The next 15 days is a rapid growth period (exponential phase) when methane production increased sharply. Then it gradually stabilized in the last period (plateau phase). However, methane production from pretreated coal increased rapidly after 20 days of cultivation when methane production from raw coal was almost stable, resulting in higher methane production. It was more likely that the bioavailability of coal increased after NaOH treatment mainly by converting previously refractory compounds in coal into biousable organic matter instead of generating new small molecular compounds that was easy to biodegrade, because the methane production curves in the earlier stages before and after treatment overlapped.

### Effects of pretreating time on methane production

**Fig 2:**
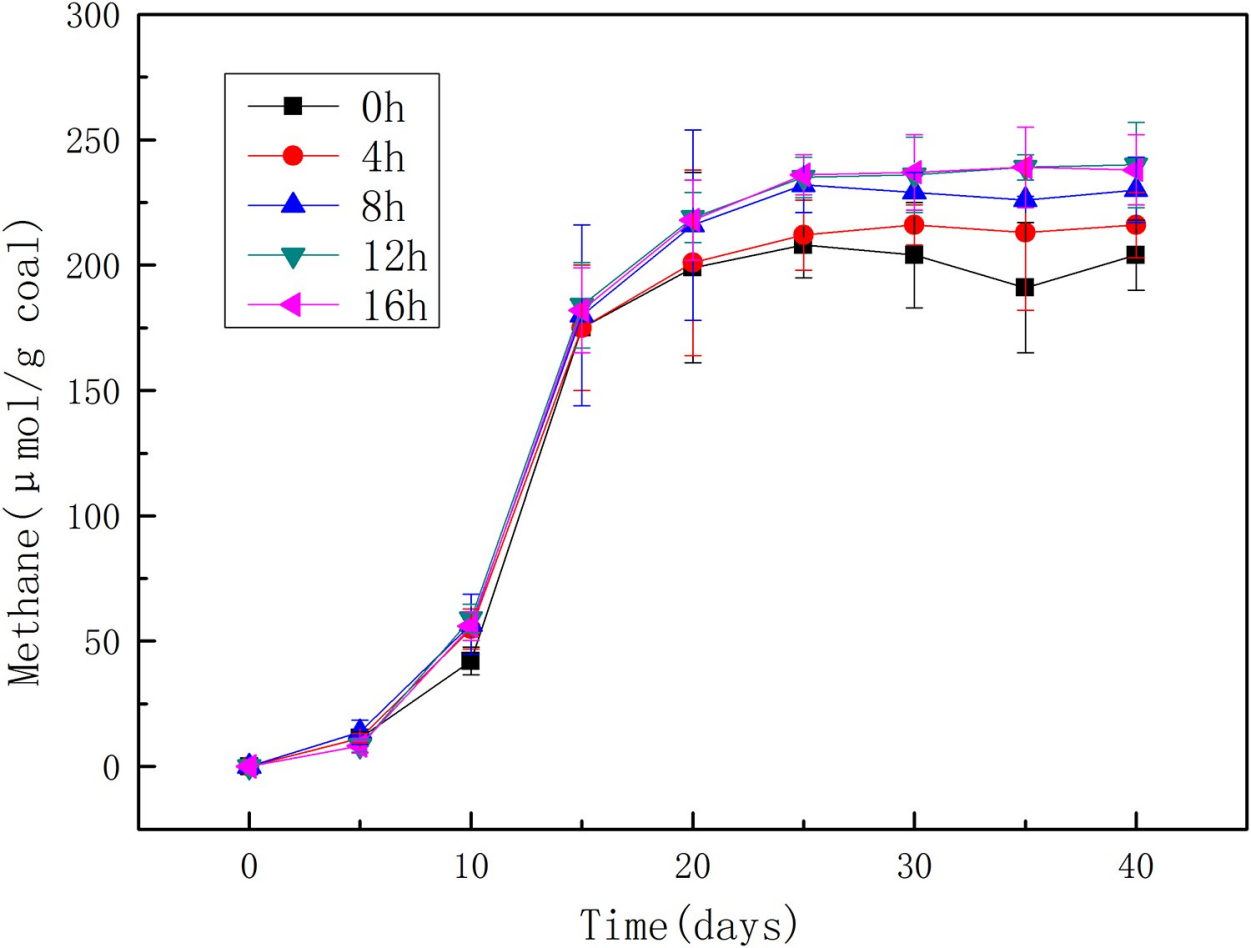
Methane productions from coal pretreated by 1.5 M NaOH for 0 h, 4 h, 8 h, 12 h, and 16 h.

The effects of pretreating time on methane production were analyzed under 1.5M NaOH which showed the most promising treatment concentration on methane production. The methane production from coal treated for 0 h, 4 h, 8 h, 12 h, and 16 h was shown in Fig 2. Similarly, different pretreating time significantly affected the methane yield but the shapes of the cumulative methane production curves did not change. Methane productions in the cultivations with coal treated by NaOH were all larger than that in the cultivations with raw coal (0 h). Methane production increased along with the increase of treating time, while almost the same methane yield was observed in the cultivations with coal pretreated for 12 h and 16 h which also represented the largest methane yield. Specifically, 216 μmol/g coal and 230 μmol/g coal were the largest methane yield from coal pretreated for 4 and 8 h, which were 5.88% and 12.75% respectively higher than that from the raw coal. Both coal pretreated for 12 and 16 h produced methane with 240 μmol/g coal, which increased methane production by about 17.65% compared with the raw coal. Based on these results, 12 h would be the best time for NaOH pretreatment. More treatment time with 1.5 M NaOH had less effect on methane production, suggesting the effect of NaOH on coal structure was no longer enhanced even if treating continued. It seems that NaOH treatment was more mild by comparing with H_2_O_2_ treatment which would resulted in less biodegradable compounds left in coal by over oxidation.

Same as the observation in the experiment of different concentrations, the methane production curves for different NaOH treating time almost overlapped in the early stage (before 15^th^ day). Then, these treatments divided into two groups based on methane production. One included whose treating time was longer than 8 h and methane production was higher. The other was those treating time was shorter than 8 h. As reported previously that alkali treatment could break, disrupt, and reform various interaction forces and entanglements between coal macromolecules [25], NaOH pretreatment may improve the bioavailability of coal through weakening or disrupting the interactions between coal macromolecules or between organic molecules and metal ions in coal, resulting in both concentration and time mainly affect the highest methane production but not methane production trend.

### Changes of coal structure by treating with 1.5 M NaOH for 12 has revealed by FTIR

**Fig 3:**
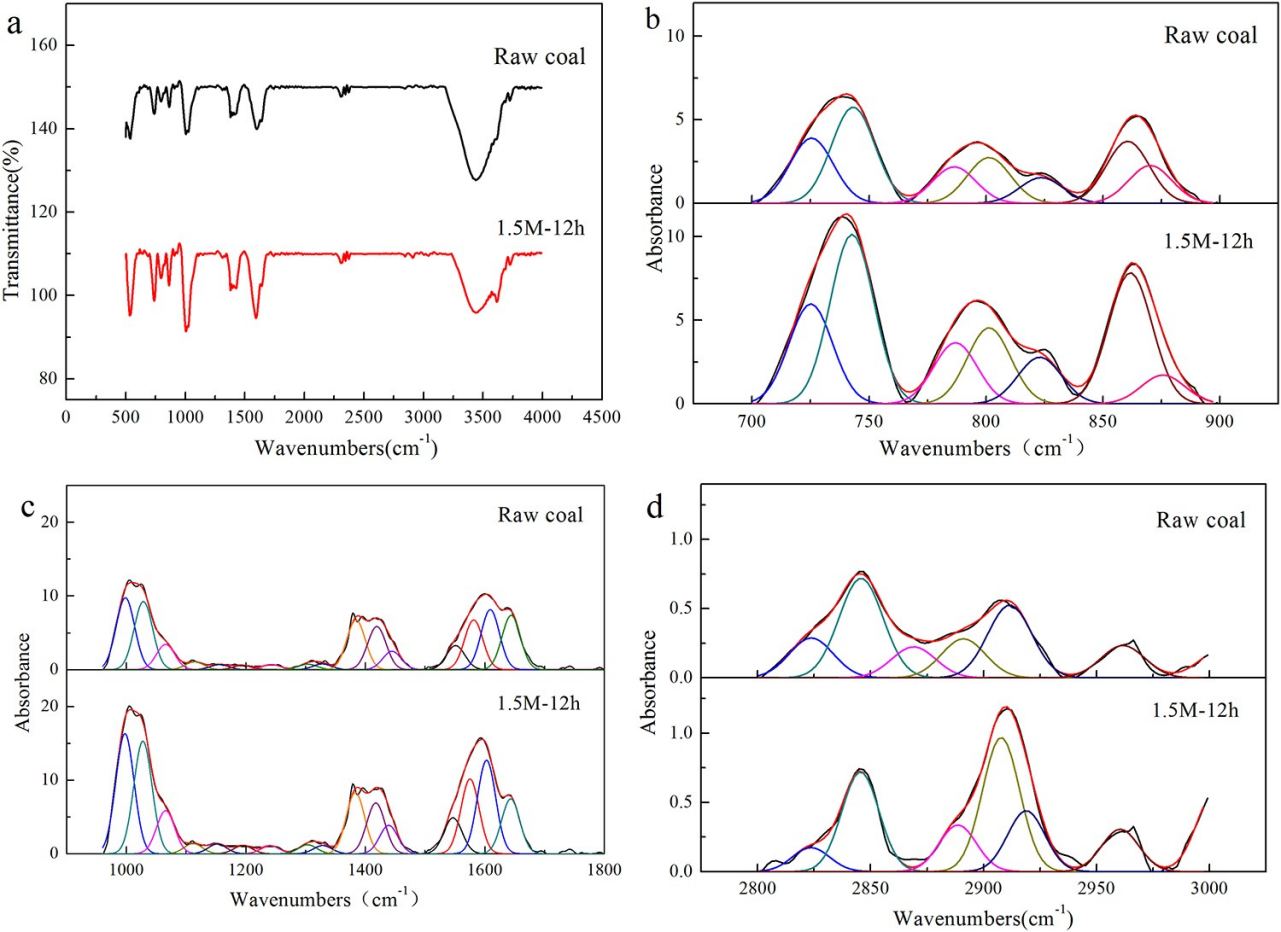
The FTIR spectrograms and curve-fitting peaks of coal samples untreated and treated with 1.5 M NaOH for 12 h. (a) Original FTIR spectrograms; (b) Curve-fitting peaks of aromatic functional groups; (c) Curve-fitting peaks of oxygen-containing functional groups; (d) Curve-fitting peaks of aliphatic functional groups.

The FTIR spectrograms of coal samples untreated and treated with 1.5 M NaOH for 12 h were shown in Fig 3a. Due to that each peak in original spectrograms commonly included several functional groups, a curve-fitting analysis method was employed to separate the original spectrograms, to analyze the detailed FTIR characteristics of raw coal and coal treated with NaOH. The selected regions of FTIR spectrum were mainly divided into three parts to fit peaks using curve-fitting software: 700–900 cm^-1^ (aromatic functional group), 1000–1800 cm^-1^ (oxygen-containing functional group), and 2800–3000 cm^-1^ (aliphatic functional group) [36]. The fitting curves of coal samples untreated and treated with 1.5 M NaOH for 12 h were shown in Fig 3b, 3c and 3d, and the correlative fitting parameters were shown in S1–S3 Tables including the height, center, width, area, and area percent of each peak. The fitting correlation coefficient r^2^ of samples are all over 0.99.

Seven peaks were fitted in the region of 700–900 cm^-1^ (Fig 3b). The attribution of each peak to a functional group was referenced to the previous literatures [36–38]. There were four substitution modes of benzene rings found in the coal sample. Among them, 2 substitutions and 5 substitutions were dominant according to the peak area. In addition to aromatic substances, alkanes side rings (CH)_n_ at 730 cm^-1^ were also observed in this region. There were some slight changes detected in aromatic compounds determined in this region caused by NaOH pretreatment (S1 Table), suggesting that NaOH could hardly act on the aromatic compounds in coal and only slightly act on the aromatic substituent groups, which was different from the H_2_O_2_ pretreatment that aromatic parts of coal were changed significantly [39]. In addition, it seemed that NaOH could act on the alkanes side rings in coal which decreased from 17.6% to 16.25%.

The infrared spectra of the studied coal were complex in the region of 1000–1800 cm^-1^, which was mainly composed of oxygen-containing functional groups and the vibration information of aromatic C = C, -CH_3_, -CH_2_, CH_3_-Ar, and so on. A total of 16 bands were detected which was in agreement with the literature elaborated by other authors [40,41]. The fitting diagram and specific data were shown in Fig 3c and S2 Table. After treated with NaOH, the intensity and area ratio of absorption peaks of C = O stretching vibration at 1644 cm^-1^ decreased from 10.92% to 7.54%. Meanwhile, the increase of intensity and area ratio of absorption peaks representing C-O alcohols, ethers, and phenols in the range of 1000–1300 cm^-1^ was observed from 24.30% to 27.58%. In addition, the area ratios of the absorption band assigned to the aromatic C = C were almost not changed, which was consistent with the results of aromatic functional group that the aromatic structure of coal was barely affected by NaOH treatment.

The amount of aliphatic substances in anthracite was small as a low-intensity band at 2800–3000 cm^-1^ was detected as shown in Fig 3d which mainly contained -CH, -CH_2_, and -CH_3_. The curve-fitting analysis revealed the presence of six absorption peaks which was consistent with the previous literatures [37,41]. After treated with NaOH, -CH_2_ functional groups locating at 2850 cm^-1^ and 2916 cm^-1^ which were dominant in raw coal accounting for about half of the total area, decreased to 36.95%. The symmetric and asymmetric stretching vibrations of aliphatic -CH_3_, which were respectively found at 2869 cm^-1^ and 2962 cm^-1^, increased to 20.47%. The infrared spectral structure parameter CH_2_/CH_3_ is generally believed to characterize the length and branching degree of the aliphatic side chain in samples. The larger the value of this parameter are, the longer the aliphatic chain is and the less the branching degree of the aliphatic chains. Here, the parameters of untreated and pretreated coal with 1.5 M NaOH for 12 h were 2.25 and 1.44, respectively, indicating that NaOH pretreatment broke the aliphatic chains in coal mainly resulting in high branching degree of aliphatic chains [42].

### Effect of NaOH pretreatment on the microcrystalline structure of coal

**Fig 4:**
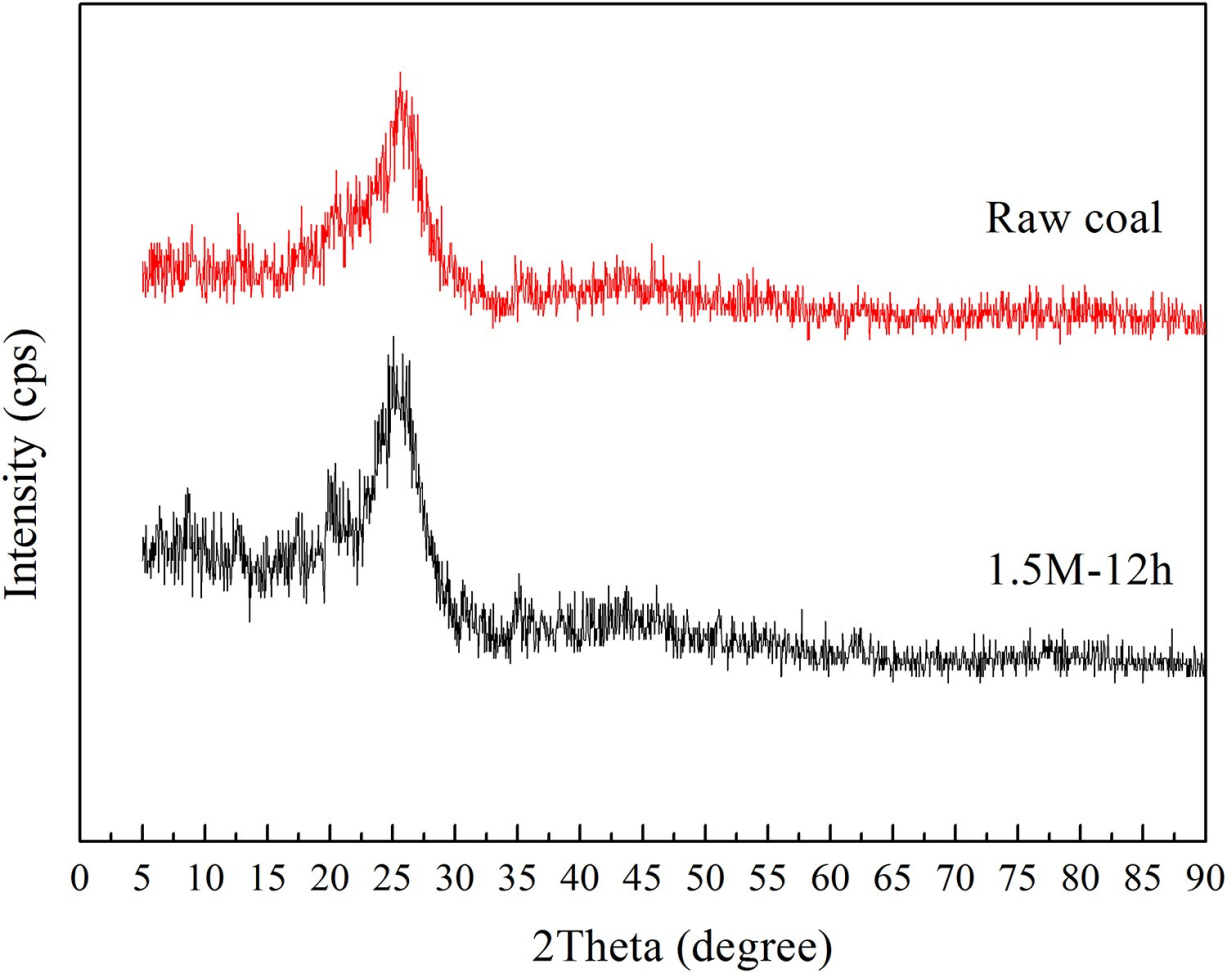
XRD diffraction patterns of raw coal and coal treated with 1.5 M NaOH for 12 h.

The XRD diffraction diagrams of the raw coal and residual coal pretreated with 1.5 M NaOH for 12 h were shown in Fig 4. The coal samples containing graphite-like structures were demonstrated by an obvious (002) peak at ~ 26° and a weak (100) peak at ~ 43°. The calculated structural parameters of all samples were shown in Table 1 including interlayer spacing (d_002_), diameter (L_a_), stack height (L_c_) of crystalline structure, the number of stacking layer of aromatic carbon (N), and the aspect ratio (L_a_/L_c_).

**Table 1:** The structural parameters of raw coal and coal samples treated with 1.5 M NaOH for 12 h revealed by XRD analysis.

sample	d_002_/Å	L_c_/Å	L_a_/Å	N	L_a_/L_c_
raw coal	0.3520	1.80	9.45	5.11	5.25
1.5M-12h	0.3562	1.66	5.61	4.66	3.38

After treated with NaOH, the interlayer spacing (d_002_) increased from 0.35 to 0.36 Å. The L_a_ and L_c_ of aromatic crystal decreased from 9.45 to 5.61 Å and from 1.80 to 1.66 Å, respectively. The number of stacking layer of aromatic carbon in coal pretreated with NaOH decreased from 4.66 compared with raw coal at 5.11, suggesting the crystalline layers became thinner. And the aspect ratio reduced from 5.25 to 3.38 Å, indicating that the decreased rate of L_a_ was greater than the decreased rate of L_c_. The d_002_ value of pretreated coal sample was higher than that of raw coal. Thus, NaOH pretreatment facilitated coal biodegradation by decreasing the microcrystalline structure of coal which was in accordance with methane production and FTIR spectroscopy analysis.

### Organics released from coal after treated with 1.5 M NaOH for 12 h

The organic components of filtrate obtained after NaOH pretreatment under 1.5 M for 12 h were shown in Table 2. Some of organics in coal were released to liquid under the treatment of NaOH. Ethers and aromatic hydrocarbons were dominant accounting for 42.84% and 38.20%, respectively. Esters, alcohols, phenols, ketones, and alkenes were also detected with proportions of 7.36%, 4.99%, 4.47%, 1.68%, and 0.47%, respectively. The detection of organics in filtrate suggested that NaOH pretreatment did act on the coal structure. And the reaction liquid obtained after treatment also were potential to contribute to methane production as the carbon number of these organics were mostly less than 10 and they could be utilized by microorganisms [43].

**Table 2:** The organic composition of filtrate after coal treatment with 1.5 M NaOH for 12 h revealed by GC-MS analysis.

Compounds	Retention time	Molecular formula	Percentage
p-Xylene	4.384	C8H10	15.54%
1,3,5-Trioxane	4.696	C3H6O3	15.62%
1,3,5-Trioxane	4.825	C3H6O3	27.22%
o-Xylene	4.965	C8H10	19.82%
3-Penten-1-ol, 2-methyl-	6.901	C6H12O	4.99%
Benzene, 1-methyl-3-(1-methylethyl)-	8.862	C10H14	0.89%
Benzene, 1,2,3,5-tetramethyl-	10.543	C10H14	1.15%
p-Cymene	11.991	C10H14	0.79%
Azulene	18.705	C10H8	0.47%
Phenol, 2,6-bis(1,1-dimethylethyl)-4-methyl-, methylcarbamate	23.364	C17H27NO2	0.70%
Phenol	25.839	C6H6O	2.96%
2,4-Di-tert-butylphenol	32.865	C14H22O	0.81%
5-Oxotetrahydrofuran-2-carboxylic acid, ethyl ester	36.232	C7H10O4	7.36%
2(3H)-Furanone, dihydro-4-hydroxy-	38.681	C4H6O3	1.68%

**Fig 5:**
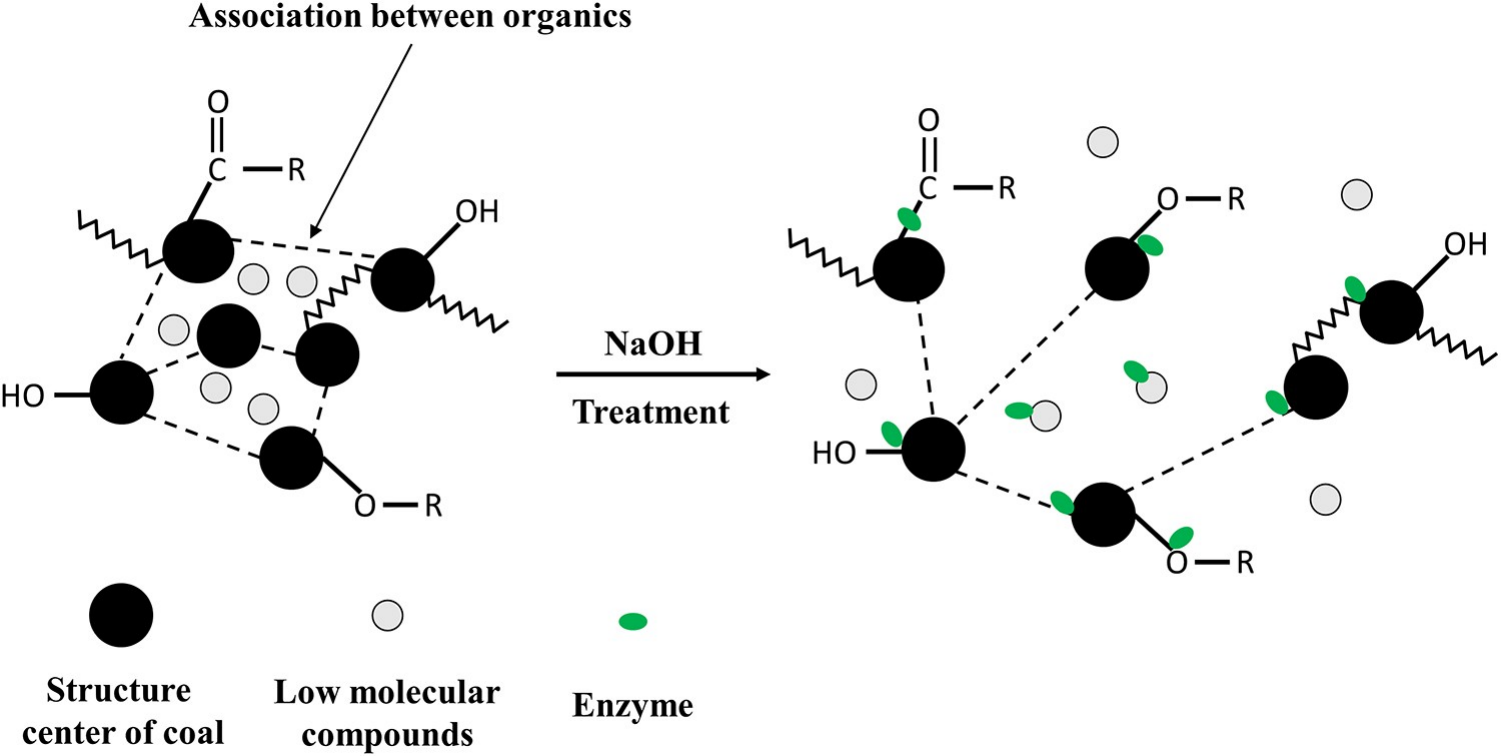
Schematic mechanism of NaOH pretreatment of coal to enhance biomethane production modified from Liu et al (2015) [46].

It is noted that there were 13 organic compounds detected which was much less than that found after H_2_O_2_ pretreatment [16]. And the weight of residual coal was reduced to 1.88 g compared with raw coal with a mass of 2.00 g which was 11% weight loss. Most of the organics in filtrate were more likely to be dissolved by NaOH as it was reported that lignin, the important components in coal, was removed from agricultural and plant wastes after pretreated with NaOH [44,45]. And the association between organics in coal would also be disrupted by NaOH treatment [44] resulting in such phenomena that the multi-substituted aromatics slightly reduced while the low-substituted aromatics increased a little, the number of carbonyl groups decreased while the percentages of C-O in alcohols and aromatic ethers increased, the branching degree of aliphatic chain increased, and the microcrystalline structure of coal decreased. Lower substituted aromatics would facilitate the interaction between coal and microbe, provide more contact area for functional microorganisms. The increase in C-O in alcohols ethers and aromatic ethers would provide more acting sites for microbes because C-O bonds were more susceptible to microbial attack. The increased branching degree of the aliphatic chain would also provide more acting sites for methanogens as methylotrophic methanogens could use -CH_3_ to generate methane. And the possible mechanism of NaOH pretreatment on coal structure was proposed (Fig 5) according to the above results. Further analysis was needed and in progress to verify the proposed mechanism.

## Conclusions

Coal is a complex mixture of organic and inorganic substances, and the organic matter in coal is mainly composed of a variety of complex polymers connected by bridging bonds, which makes it difficult for microorganisms to directly utilize it as substrates, especially for anthracite. In this paper, NaOH was used to pretreat anthracite to facilitate the biodegradation of coal to enhance methane production. The results showed that the raw coal can be degraded to produce methane by an anaerobic microflora with a yield of about 204 μmol/g coal. NaOH treatment has shown to increase methane yield with respect to treatment dosage and time. Pretreatment of coal using 1.5 M NaOH for 12 h was the optimal condition for methane production when the maximum amount of methane production (240 μmol/g coal) increased by 17.65%. NaOH mainly reduced the carbonyl groups and microcrystalline structure of coal, increased the C-O in alcohols and aromatic ethers and branching degree of aliphatic chain by removing some organics from coal and disrupting the association between organics in coal. These changes facilitated the interaction between coal and microbe and improved the bioavailability of coal, thus enhancing the methane production. These results suggested that NaOH was an effective solution for pretreating coal to enhance biogenic methane production.

## Supporting information

S1 Table The curve-fitting parameters of aromatic functional groups of raw coal and coal samples treated with 1.5 M NaOH for 12 h (700-900cm^-1^). (DOCX)

S2 Table The curve-fitting parameters of oxygen-containing functional groups of raw coal and coal samples treated with 1.5 M NaOH for 12 h (1000-1800cm^-1^). (DOCX)

S3 Table The curve-fitting parameters of aliphatic functional groups of raw coal and coal samples treated with 1.5 M NaOH for 12 h (2800-3000cm^-1^). (DOCX)
