# Supplementary material for: The effect of NaOH pretreatment on coal structure and biomethane production
Source: PLoS One. 2020 Apr 15;15(4):e0231623. doi: 10.1371/journal.pone.0231623 (PMC7159192; doi:10.1371/journal.pone.0231623)
Supplement: S1 Table — (DOCX) [file pone.0231623.s001.docx]

S1 Table The curve-fitting parameters of aromatic functional groups of raw coal and coal samples treated with 1.5 M NaOH for 12 h (700-900cm^-1^)

| Peak | sample | Amplitude | Center | Int Area | % Area | Assignment |
| --- | --- | --- | --- | --- | --- | --- |
| 1 | Raw coal | 3.90 | 725.41 | 90.14 | 17.62 | alkanes side rings[(CH)n,n>4] |
|  | 1.5M-12h | 5.98 | 725.09 | 138.84 | 16.25 |  |
| 2 | Raw coal | 5.76 | 743.21 | 133.43 | 26.08 | Aromatics with 2 substitutions |
|  | 1.5M-12h | 10.14 | 742.71 | 236.40 | 27.66 |  |
| 3 | Raw coal | 2.18 | 786.42 | 50.59 | 9.89 | Aromatics with 3 substitutions |
|  | 1.5M-12h | 3.66 | 786.95 | 85.35 | 9.99 |  |
| 4 | Raw coal | 2.74 | 801.26 | 63.48 | 12.41 | Aromatics with 4 substitutions |
|  | 1.5M-12h | 4.55 | 801.29 | 106.14 | 12.42 |  |
| 5 | Raw coal | 1.54 | 823.62 | 35.67 | 6.97 | Aromatics with 4 substitutions |
|  | 1.5M-12h | 2.79 | 822.98 | 65.11 | 7.62 |  |
| 6 | Raw coal | 3.71 | 860.59 | 85.97 | 16.80 | Aromatics with 5 substitutions |
|  | 1.5M-12h | 7.84 | 861.67 | 182.80 | 21.39 |  |
| 7 | Raw coal | 2.27 | 870.20 | 52.42 | 10.24 | Aromatics with 5 substitutions |
|  | 1.5M-12h | 1.72 | 875.67 | 39.92 | 4.67 |  |
