# Supplementary material for: The effect of NaOH pretreatment on coal structure and biomethane production
Source: PLoS One. 2020 Apr 15;15(4):e0231623. doi: 10.1371/journal.pone.0231623 (PMC7159192; doi:10.1371/journal.pone.0231623)
Supplement: S2 Table — (DOCX) [file pone.0231623.s002.docx]

S2 Table The curve-fitting parameters of oxygen-containing functional groups of raw coal and coal samples treated with 1.5 M NaOH for 12 h (1000-1800cm^-1^)

| Peak | sample | Amplitude | Center | Int Area | % Area | Assignment |
| --- | --- | --- | --- | --- | --- | --- |
| 1 | Raw coal | 9.76 | 998.42 | 373.61 | 14.37 | Si-O |
|  | 1.5M-12h | 16.32 | 997.65 | 618.54 | 16.40 |  |
| 2 | Raw coal | 9.25 | 1028.55 | 356.69 | 13.71 | alkyl ethers, Si-O |
|  | 1.5M-12h | 15.30 | 1027.76 | 583.87 | 15.48 |  |
| 3 | Raw coal | 3.42 | 1065.62 | 131.66 | 5.06 | v C-O sec. alcohols |
|  | 1.5M-12h | 5.84 | 1065.69 | 223.09 | 5.91 |  |
| 4 | Raw coal | 1.04 | 1114.21 | 40.26 | 1.55 | v C-O, tert. alcohols, ethers |
|  | 1.5M-12h | 1.37 | 1111.69 | 52.47 | 1.39 |  |
| 5 | Raw coal | 0.68 | 1156.09 | 26.32 | 1.01 | v C-O phenols, ethers |
|  | 1.5M-12h | 1.32 | 1152.30 | 50.40 | 1.34 |  |
| 6 | Raw coal | 0.57 | 1194.13 | 22.00 | 0.85 | v C-O phenols, ethers |
|  | 1.5M-12h | 1.11 | 1195.24 | 42.52 | 1.13 |  |
| 7 | Raw coal | 0.69 | 1242.73 | 26.50 | 1.02 | v C-O and δ OH, phenoxy structures, ethers |
|  | 1.5M-12h | 1.09 | 1240.78 | 41.76 | 1.11 |  |
| 8 | Raw coal | 0.74 | 1303.70 | 28.57 | 1.10 | v C-O in aryl ethers |
|  | 1.5M-12h | 1.21 | 1303.48 | 46.35 | 1.23 |  |
| 9 | Raw coal | 0.77 | 1327.96 | 29.75 | 1.14 | δ s. CH2-C = O |
|  | 1.5M-12h | 1.11 | 1328.74 | 42.53 | 1.13 |  |
| 10 | Raw coal | 6.73 | 1384.56 | 259.35 | 9.97 | δ s. CH3-Ar, R |
|  | 1.5M-12h | 8.31 | 1383.55 | 317.15 | 8.41 |  |
| 11 | Raw coal | 5.85 | 1419.36 | 225.67 | 8.68 | aromatic C = C |
|  | 1.5M-12h | 6.89 | 1417.67 | 263.20 | 6.98 |  |
| 12 | Raw coal | 2.50 | 1444.65 | 96.32 | 3.70 | δ as. CH3-, CH2- |
|  | 1.5M-12h | 3.85 | 1439.26 | 147.09 | 3.90 |  |
| 13 | Raw coal | 3.27 | 1551.21 | 126.09 | 4.85 | aromatic C = C |
|  | 1.5M-12h | 4.87 | 1546.76 | 185.97 | 4.93 |  |
| 14 | Raw coal | 6.75 | 1581.42 | 259.99 | 10.00 | aromatic C = C |
|  | 1.5M-12h | 10.16 | 1575.23 | 387.76 | 10.28 |  |
| 15 | Raw coal | 8.14 | 1609.17 | 313.89 | 12.07 | aromatic C = C |
|  | 1.5M-12h | 12.70 | 1602.93 | 484.94 | 12.86 |  |
| 16 | Raw coal | 7.37 | 1644.19 | 284.13 | 10.92 | conjugated C = O |
|  | 1.5M-12h | 7.45 | 1643.32 | 284.53 | 7.54 |  |
