# Supplementary material for: The effect of NaOH pretreatment on coal structure and biomethane production
Source: PLoS One. 2020 Apr 15;15(4):e0231623. doi: 10.1371/journal.pone.0231623 (PMC7159192; doi:10.1371/journal.pone.0231623)
Supplement: S3 Table — (DOCX) [file pone.0231623.s003.docx]

S3 Table The curve-fitting parameters of aliphatic functional groups of raw coal and coal samples treated with 1.5 M NaOH for 12 h (2800-3000cm^-1^)

| Peak | Sample | Amplitude | Center | Int Area | % Area | Assignment |
| --- | --- | --- | --- | --- | --- | --- |
| 1 | Raw coal | 0.29 | 2823.73 | 6.96 | 12.29 | CH vibration in aldehydes |
|  | 1.5M-12h | 0.17 | 2823.62 | 3.57 | 5.50 |  |
| 2 | Raw coal | 0.72 | 2845.77 | 17.52 | 30.93 | aliphatic CH2 symmetric stretching vibration |
|  | 1.5M-12h | 0.72 | 2845.50 | 14.87 | 22.93 |  |
| 3 | Raw coal | 0.22 | 2869.05 | 5.46 | 9.64 | aliphatic CH3 symmetric stretching vibration |
|  | 1.5M-12h | 0.34 | 2888.65 | 6.96 | 10.73 |  |
| 4 | Raw coal | 0.28 | 2891.09 | 6.87 | 12.14 | aliphatic CH stretching vibration |
|  | 1.5M-12h | 0.97 | 2907.77 | 20.02 | 30.87 |  |
| 5 | Raw coal | 0.53 | 2911.60 | 12.79 | 22.59 | aliphatic CH2 asymmetric stretching vibration |
|  | 1.5M-12h | 0.44 | 2918.89 | 9.09 | 14.01 |  |
| 6 | Raw coal | 0.23 | 2961.71 | 5.69 | 10.05 | aliphatic CH3 asymmetric stretching vibration |
|  | 1.5M-12h | 0.31 | 2960.52 | 6.32 | 9.74 |  |
